# Natural Killer Cell Expansion with Autologous Feeder Layer and Anti-CD3 Antibody for Immune Cell Therapy of Hepatocellular Carcinoma

**DOI:** 10.31557/APJCP.2019.20.12.3797

**Published:** 2019

**Authors:** Faezeh Hosseinzadeh, Jafar Ai, Somayeh Ebrahimi-Barough, Iman Seyhoun, Abbas Hajifathali, Samad Muhammadnejad, Fatemeh Hosseinzadeh, Mahdi Shadnoush, Farnaz Dabiri Oskouei, Javad Verdi

**Affiliations:** 1 *Department of Tissue Engineering and Applied Cell Sciences,School of Advanced Technologies in Medicine, *; 4 *Cell-Based Therapies Research Center, Digestive Diseases Research Institute, *; 5 *Dentistry Faculty, Tehran University of Medical Sciences, *; 3 *Taleghani Bone Marrow Transplantation Center,Taleghani Hospital, *; 6 *Department of Clinical Nutrition, Faculty of Nutrition and Food Technology, Shahid Beheshti University of Medical Sciences, Tehran, *; 2 *Department of Tissue Engineering, Qom University of Medical Sciences, Qom, *; 7 *Department of Stem Cell Therapy, Tabriz Valiasr Hospital, Tabriz, Iran. *

**Keywords:** Natural Killer cell expansion, immune cell therapy, Hepatocellular Carcinom

## Abstract

**Background:**

one of the promising approaches for treatment of some cancers is adoptive cell therapy using natural killer (NK) cells. Various methods have been investigated for ex vivo expansion of NK cells in large-scale, but most of them involved cancer or genetically modified cells as feeder layer and also some of them have the risk of T cell contamination and graft-versus-host disease (GVHD).

**Method:**

In this study, irradiated autologous peripheral blood mononuclear cells (PBMCs) as feeder layer with an anti-CD3 monoclonal antibody (mAb) were used. For activation and expansion of NK cells, human recombinant IL2 and IL15 were used. After co-culturing of expanded NK cells (eNKC) and isolated NK cells (iNKC) with hepatocellular carcinoma (HCC) cells, the viability of eNKC in compared to iNKC were analyzed by CCK-8 assay and degranulation of NK cells after co-culturing was assayed by measuring CD107a expression. Enzyme-Linked Immunosorbent Assay (ELISA) assay was used for the ability of NK cells to secretion of IFN-γ (interferon-γ) and TNF-α (Tumor Necrosis Factor-α) after co-culture with HCC cells. Real Time PCR analysis was used for expression of human Perforin and Granzyme B genes in the NK cells exposed to target HepG2 cells.

**Result:**

This method strongly expanded highly purified NK cells with powerful cytotoxicity against HCC cells. The expanded NK cells showed high level of expression of degranulation marker and human Perforin and Granzyme B genes, and also was secreted larger amounts of TNF-α and IFN-γ compared with fresh isolated NK cells.

**Conclusion:**

we proposed an effective method for expansion of cytotoxic NK cells using irradiated autologous PBMC as feeder layer for more successful transfer of allogeneic NK cell in immuno cell therapy of HCC.

## Introduction

Up to 20 percent of human peripheral blood lymphocytes was composed NK cells that defined as CD3−CD56+ cells (Cooper et al., 2001). The cytotoxicity of NK cell is regulated by some inhibitory and stimulatory signaling pathways (Cho and Campana, 2009). The main effective function of NK cells is induction of apoptosis via expression of death-receptor ligands, secretion of cytoplasmic granules (such as Perforin and Granzyme), antibody-dependent cellular cytotoxicity (ADCC) or by secretion of cytokines such as TNF-α and IFN-γ (Cooper et al., 2001; Srivastava et al., 2008). One of the inhibitory mechanisms for NK cell activity is increasing the expression of surface major histocompatibility complex (MHC) class I in non-tumor target cells that can prevent from GVHD after allogenic NK cells transplantation (Ljunggren and Karre, 1990; Ljunggren and Malmberg, 2007). Adoptive NK cells are suitable candidate cells for immune cell therapy of cancer, but the yield of isolated NK cells from peripheral blood mononuclear cell (PBMC) is not always enough for performing multiple clinical applications (Klingemann, 2015; Chabannon et al., 2016). Therefore, different studies have investigated on the ex vivo expansion of NK cells from various sources such as PBMC, bone marrow, cord blood and embryonic stem cells (Cheng et al., 2013; Koepsell et al., 2013). In these efforts, cancer cell-based feeder cells such as genetically modified K562 cells (Fujisaki et al., 2009; Gong et al., 2010), or Epstein–Barr virus-transformed lymphoblastoid cell lines (Berg et al., 2009) have been used. In some methods, it is suggested to expansion of NK cell from whole peripheral blood lymphocytes after inhibition of T cells proliferation but some T cells may be remain after NK cell expansion (Alici et al., 2008; Fujisaki et al., 2009; Klingemann and Martinson, 2004; Peng et al., 2004). Therefore, some studies used irradiated autologous PBMC with the cytokines and mABs, as feeder layer, for NK cell expansion (Ahn et al., 2013; Lee et al., 2017). Many cytokines, have been applied for expansion and activation of NK cells such as IL-21, -18, -15, -12 and -2(Cooper et al., 2009; Romee et al., 2014).

Hepatocellular carcinoma (HCC) is one of the most common cancer and second cause of death from cancer in the world (Jemal et al., 2011). Despite of several effective treatment options of this cancer, only early-stage tumors can be treated (Huynh, 2010; Lin et al., 2012). Only less than 20% of HCC patients are eligible for such treatments, because of diagnosis of this disease at an advanced stage, limitation of liver donors and heterogeneity of HCC tumor (Davila et al., 2012). Immune cell therapy of HCC with NK cells highly considered in the last decades to become the new standard of care for advanced hepatocellular carcinoma (HCC) (Tian et al., 2013). Despite the fundamental role of NK cells in the hepatic regeneration, the frequency and effecter function of NK cells have been impaired over the progression of HCC (Sun et al., 2015). Therefore, there are many strategies to overcome exhaustion or dysfunction of NK cells for HCC treatment (Hosseinzadeh et al., 2018).

Here, we investigated an ex vivo method for activation and expansion of NK cells using autologous feeder layer and anti-CD3 antibody. In the next step, we examined the efficacy and cytotoxicity of activated and expanded NK cells (eNKC) against HCC cell line, and compared with that of fresh isolated NK cells (iNKC), in vitro.

## Materials and Methods

*HCC cell culture*

Human HCC cell line (HepG2) was purchased from the Iranian Biological Resource Center (IBRC) and maintained in high glucose DMEM media (Gibco D5796, USA), supplemented with 10% heat inactivated fetal bovine serum (FBS, Gibco, USA), 100 U/ml penicillin and 100 μg/ml streptomycin in standard conditions of incubator at 37°C, in a 95% humidified atmosphere and 5% CO_2_. 

*Isolation of NK Cells and Flow Cytometry Assay*

Primary NK cells were isolated from peripheral blood mononuclear cells of 3 healthy donors under the approval of the Institutional Ethical Committee of Tehran University of Medical Sciences (TUMS). PBMCs were isolated from peripheral whole blood by density gradient centrifugation on Ficoll-Hypaque (GE Healthcare, GE17-5442-02 Sigma, Sweden). NK cells were isolated from the buffy coats using magnetically activated cell sorting (MACS) system by NK Cell Isolation Kit and MACS columns via negative selection (Miltenyi Biotec, Germany). Purity of the isolated human NK cells (CD3^-^, CD56^+^) was determined using Attune NxT acoustic focusing flow cytometer and antibodies against CD56 (PE, EXBIO, Czech Republic), CD3 (FITC, Beckman Coulter, US). On average, the isolated NK cell purity was more than 95% in all steps of experiment.

*NK cell expansion using autologous feeder layer, OKT3 and cytokines*

After isolation of NK cells from PBMC, the NK-depleted cells obtained during the MACS procedure (CD3^+^ or CD56^-^ cells) were γ-irradiated (2500 cGy), and cocultured with NK cells for preparation of suitable environment as autologous feeder cells. Purified NK cells with MACS separation were cultured in SCGM medium (CellGenix, Freiburg, Germany), containing 10% FBS, 1% Penestrep, with irradiated autologous PBMC as feeder cells at a ratio of 1:10 (for example 100,000 NK cells with 1000,000 feeder cells in 1cc media), in standard conditions of incubator at 37°C in a 95% humidity and 5% CO_2_. Media was supplemented with human recombinant interleukin-2 (hrIL-2) (eBioscience™, US) (1,000 IU/mL), hrIL-15 (eBioscience™, US) (10 ng/ml), anti-CD3 (OKT3) (Cytomatin Gene, Iran) (10 ng/ml) and human serum AB+ (5%). After 5 days, all cells were harvested and after centrifuge, the media completely changed and cells resuspended in fresh media containing rhIL-2 (1,000 U/mL) and rhIL-15 (10 ng/ml) without OKT3 and human serum AB+. In the after steps, half of the media was changed and replaced by fresh media with cytokines every 1 to 2 days for 14 days. When the cell density was high, cells were harvested and transferred to larger flasks. Purities and counts of expanded cells after separation were analyzed by trypan blue staining and flow cytometry. 

*Cell Viability Assays*

The viability of HepG2 cells evaluated by cell counting kit-8 assay (CCK-8 kit, Molecular Devices, Japan). Various effector-to-target cell count ratios of iNKC: HepG2 and eNKC: HepG2 (1:10, 1:1 and 10:1) were seeded in 96 well micro plates in triplets. CCK-8 reagent was added to each well at the same time on 24 h after co-culture of HepG2 and NK cells and incubated at 37°C and 5% CO_2_ for 2h. Absorbance was measured using a micro plate reader at 490 nm and the results were expressed as an average of three replications in each group. Fresh media was used for control groups.

*Analysis of NK cell degranulation (CD107a)*

Each type of NK cells were co-cultured with target HepG2 cells at a ratio of 1:1 at 37°C and 5% CO_2_ for 24 h and then again incubated with the FITC-conjugated anti-CD107a antibody (BD Biosciences) and Monensin (GolgiStop; BD Biosciences) for 4 h. After that, the cells were stained with FITC-conjugated anti-CD3 and PE-conjugated anti-CD56 antibodies. After 30 min, the cells were washed and analyzed by flow cytometry.

*Analysis of TNF-α and IFN-γ secretion by NK cells against HCC cells by ELISA assay*

NK cells were co-cultured with target cancer cell line HepG2 at different E:T ratios (1:10, 1:1 and 10:1) in round-bottomed 96-well plate and then were incubated for 24 h in a humidified incubator at 37°C with 5% of CO_2_. Cell-free supernatants were harvested and analyzed by appropriate ELISA kits to detect the contents of human interferon gamma (IFNG) ((ab100537), Abcam, UK) and human TNF alpha ((ab181421), Abcam, UK).

*Quantitative Real-Time PCR analysis*

After the co-culture of each of iNKC and eNKC with HepG2 cells, at different effector-to-target ratios, the supernatants were harvested and were centrifuged in order to separating NK cells. The expression levels of human Perforin and Granzyme B genes in harvesting NK cells were quantified using quantitative real-time PCR. For isolation of total RNAs, the RNeasy plus mini kit was used according to the manufacturer’s instructions (Qiagen, Germany). Then the first strand complementary DNA (cDNA) was synthesized with cDNA synthesis kit (Takara Bio, Inc., Japan). To measurement the mRNA expression levels of target genes, 10 ng cDNA (1 μl) and appropriate primers (1 μl) were mixed with Power SYBR Green PCR Master Mix (10 μl ) with diethyl pyro carbonate (DEPC) water (8 μl) in a total volume of 20μl and evaluated by using a 7500 Real-Time PCR System. The temperature adjustment was the same for all target genes; 30 s initial denaturation at 95°C, 30 s annealing at 60°C and 35 s extension at 72°C were repeated for 45 cycles. All qRT-PCR reactions were repeated in triplicate and normalized with glyceraldehyde 3-phosphate dehydrogenase (GAPDH) mRNA, which used as the internal comparator in parallel with the control sample. RT-PCR data were analyzed using the comparative Ct, 2^−ΔΔCT^, method.

*Statistical analysis*

Results are reported as mean ± SD. Differences between the two groups were calculated using Student’s t-test. Differences were considered statistically significant at p-values under 0.05.

## Results

*Autologous irradiated PBMC with anti CD3 mAb induce expansion of NK cells*

**Figure 1:**
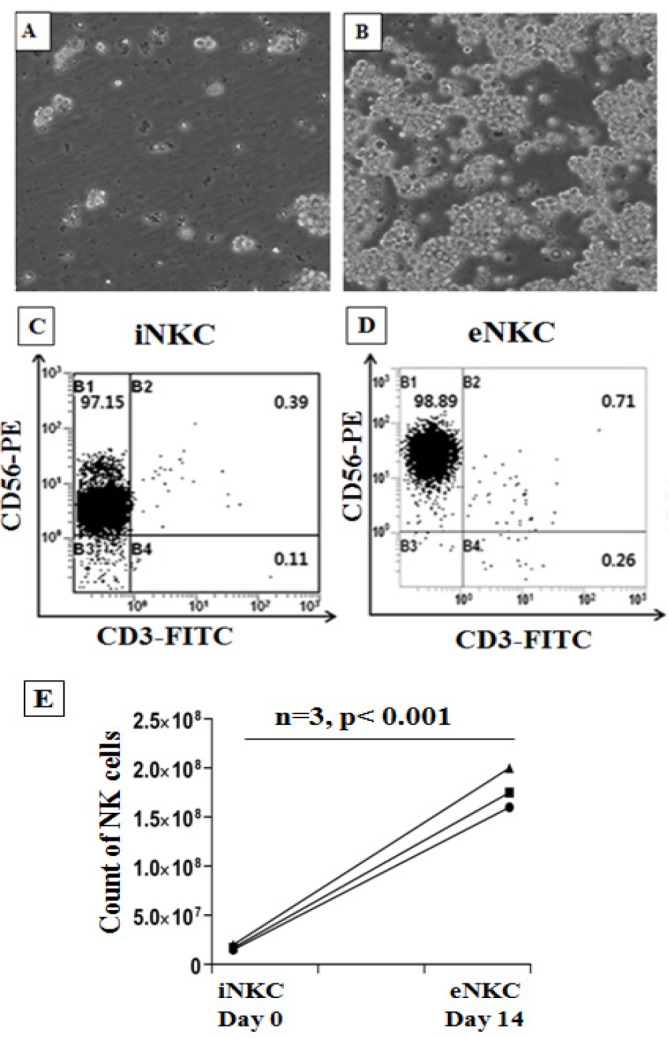
A Combination of the Anti-CD3 (OKT3) mAb with Irradiated Autologous PBMCs as Feeder Layer Strongly Induces NK Cell Expansion. A, B. The morphology of isolated and expanded NK cells at respectively day 0 and day 14. C, D. Characterization of iNKC and eNKC using human anti-CD3 and anti-CD56 by flowcytometer. E. The count of NK cells from 3 donors was determined before (day 0) and after (days 14) of NK cell expansion. Paired Student t-tests were used for statistical analysis (*** p < 0.001).

The NK cells were isolated from the buffy coats of three healthy donors using MACS method via negative selection and were characterized with flow cytometry assay. Then, the isolated NK cells were cultured at the presence of autologous irradiated PBMC as feeder layer, OKT3 mAb, rhIL-2 and rhIL-15, under specific conditions. After expansion of NK cells for 14 days, the cells were harvested and counted with tripan blue and the purity of expanded CD3-, CD56+ NK cells was determined using flow cytometer. As illustrated in Figure 1, the feeder layer with anti CD3 mAb and cytokines strongly induced the proliferation of isolated NK cells. On average, the NK cell purity was more than 95% in all steps of experiment.

*Inhibitory effect of isolated or expanded NK cells on HCC cell growth*

**Figure 2:**
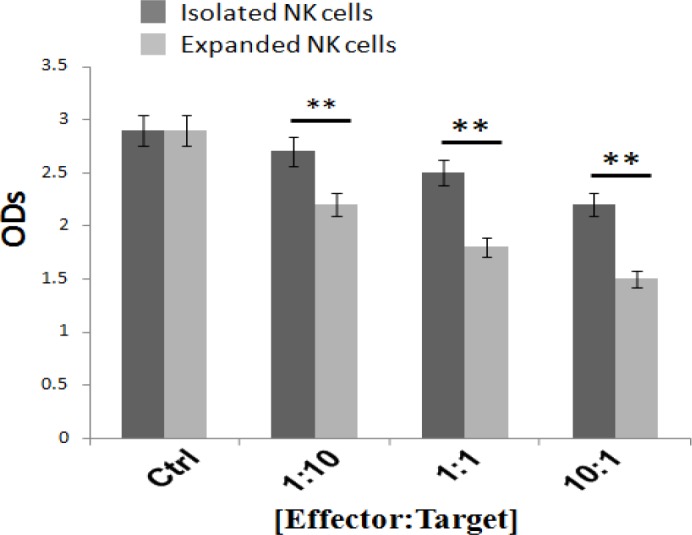
Inhibitory Effects of Isolated NK Cells and Expanded NK Cells on HepG2 Cell Growth after 24 h of Coculture, which Determined by CCK-8 Assay. All treated groups showed a significant reduction in the proliferation capacity of HepG2 cells compared to the control group. Furthermore, the inhibitory effect of expanded NK cells on HCC cell growth was significantly more than that of isolated NK cells ( * p < 0.05, ** p < 0.01, *** p < 0.001). Data are expressed as the mean±SEM of three replicates

The anti-proliferative effects of NK cells (iNKC or eNKC (after 24 h of coculture with HepG2 cells, at various E:T ratios, were determined using CCK-8 assay. Results illustrated that at different effector-to-target cell count ratios, the proliferation capacity of HepG2 cells was clearly decreased and was significantly lower than that of control groups (Figure 2). In addition, as shown in Figure 2, the anti-proliferative effect of eNKC against HCC cells, in different concentrations, was significantly more than that of iNKC (*** p < 0.001, ** p < 0.01, * p < 0.05).

*Induction of CD107a expression on expanded NK cells against HCC cells*

**Figure 3:**
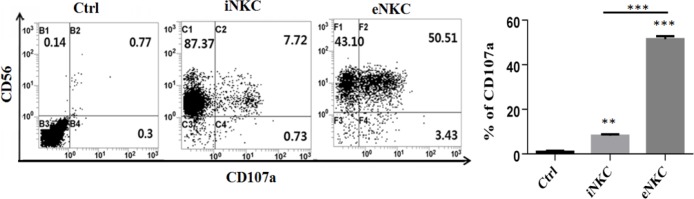
CD107a Expression was Significantly Upregulated in the Activated and Expanded NK Cells with Irradiated Autologous Feeder Cells and OKT3. CD107a analysis was performed as described in the Methods. A. Representative FCM dot plots. B. Comparison of expression levels of CD107a in iNKC and eNKC compared to Ctrl group. The degranulation level in eNKC treatment group was significantly higher than that of iNKC and Ctrl group. The assay was conducted in triplicate for each donor. Results were shown as mean ± SD, n,3. The statistical significance was determined by Student’s t-test (* p < 0.05, ** p < 0.01, *** p < 0.001).

The activity of NK cells correlates closely with expression of CD107a marker (Alter et al., 2004). We evaluated the expression level of degranulation marker CD107a in fresh isolated and also expanded NK cells under specific conditions (iNKC or eNKC). NK cells were cocultured with HepG2 cells at a ratio of 1:1 for 24 h and then were incubated in the presence of Monessen and anti-*CD107*a mAb for 4 h. Then, the supernatants were harvested and the cells in supernatant were stained with anti-CD3 and anti-CD56 mAbs and evaluated by flowcytometer. As shown in Figure 3, the little *CD107a *marker was expressed in the isolated NK cells upon contact with HepG2 cells. While, *CD107a* expression in the activated and expanded NK cells (under specific culture conditions) against HCC cells significantly increased compared to iNKC and control groups.

*Expanded NK cells significantly increased secretion of TNF-α and IFN-γ after simulation with target HCC cells*

**Figure 4.**
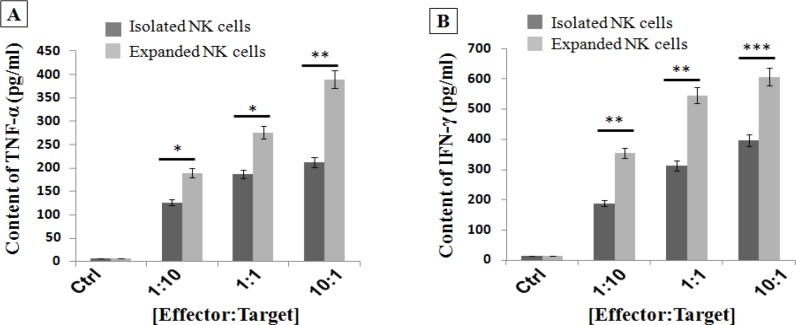
Activated and Expanded NK Cells (eNKC) under Specific Condition Significantly Increased the Secretion of A. TNF-α and B. IFN-γ after encountering target cancer cells, compared to fresh isolated (iNKC) and Ctrl group. The TNF-α and IFN-γ ELISA assays kits were performed as described in the methods. The results are shown as the average number of spots ± SD. The assay was conducted in triplicate for each donor (n, 3). The statistical significance was determined by Student’s t-test (* p < 0.05, ** p < 0.01, *** p < 0.001)

We analyzed TNF-α and IFN-γ secretion of NK cells after 24 h of co-culture with different ratios of HepG2 cells, using appropriate ELISA assay kits. As shown in Figure 4, isolated NK cells which exposed to HepG2 cells relatively secreted low amounts of TNF-α and IFN-γ, but expanded NK cells under specific culture conditions strongly increased TNF-α and IFN-γ secretion against target HepG2 cells.

*Up-regulation of Perforin and Granzyme B genes in the expanded NK cells against target HepG2 cells*

The Perforin and Granzyme B are cytoplasmic granules which are produced by activated NK cells against target cancer cells. The expression levels of human Perforin and Granzyme B genes were measured in the NK cells after simulation with HepG2 cells, at different E:T cell count ratios (1:10, 1:1 and 10:1) for 24 h, using Real-Time PCR analysis. The results indicated that these genes were significantly upregulated in the activated and expanded NK cells (eNKC) compared to isolated NK cells (iNKC) or control group. 

**Figure 5:**
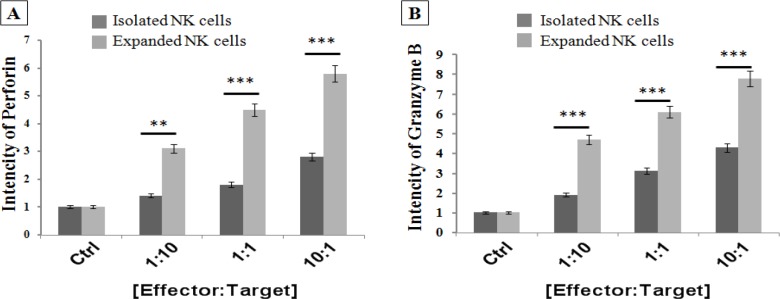
Comparison of Expression Levels of Perforin and Granzyme B Genes in Expanded NK Cells (eNKC) at the Presence of Autologous Feeder Layer and OKT3 and Isolated NK Cells (iNKC) after Stimulation with Target HCC Cells at Different E,T ratios. A significant induction was found in the expression of Perforin and Granzyme B genes in eNKC relative to that of iNKC. Expression of mRNA was detected by Real Time PCR and normalized to GAPDH; (n=3)

## Discussion

Immune-cell therapy of HCC with NK cells is an attractive approach in recent years due to high frequency and cytotoxicity of hepatic NK cells (Ishiyama et al., 2006; Tian et al., 2013) and also for unique property of NK cells faced with surface MHC-I gene expressed on the normal cells that led to inhibition of NK cells and prevention of GVHD (Ljunggren and Malmberg, 2007). The function of NK cells against tumor cells is through various mechanisms including releasing cytoplasmic granules (such as Perforin and Granzyme), secretion of various cytokines (such as IFN-γ and TNF-α), triggering death receptor-mediated apoptosis (such as FasL or TRAIL), ADCC by CD16 antigen expression on the NK cells, and also indirectly via interaction with other immune cells (Cheng et al., 2013). Progression of liver disease led to reduction, functional impairment and exhaustion of NK cells (Cai et al., 2008; Sun et al., 2015), so numerous strategies have been reported to overcome this problem which summarized in our previous study (Hosseinzadeh et al., 2018). One of the problem solving approaches is transplantation of NK cells to HCC patients, but the amount of NK cells derived from various sources is less than clinical use. Therefore, many techniques have been proposed for activation and expansion of NK cells (Cho and Campana, 2009; Ahn et al., 2013; Kamiya et al., 2016; Lee et al., 2017).

In this study, we used an improved method for activation and expansion of NK cells at present of an anti-CD3 mAb and irradiated autologous PBMCs as feeder layer, for immune cell therapy of HCC. Feeder cells provide a suitable environment for expansion of NK cells through production of soluble growth factors and direct cell-to-cell interactions (Ehmann et al., 1998). In the next step, we examined the cytotoxicity of expanded NK cells (eNKC) against HCC cells and compared with that of fresh isolated NK cells (iNKC). In this procedure, NK cells were first isolated PBMC of three healthy donors by NK isolation kit (Milteney Biotech) via negative selection and then were characterized with flowcytometer. As shown in Figure 1, the purity of isolated NK cells (CD56+, CD3-) was more than 95%. After that, NK-depleted PBMCs were γ-irradiated (2500 Gy) for effective inactivation of lymphocyte proliferation (Pelszynski et al., 1994) and cocultured with NK cells at a ratio of 1:10 (NK:feeder) in the SCGM media supplemented with OKT3 (10 ng/mL), rhIL-2 (1000 U/mL), rhIL-15 (10 ng/ml) and human serum AB+ (5%). After 5 days, fresh media without OKT3 with rhIL-2 and rhIL-15 were added every 1–2 days until day 14. The purity and yield of expanded NK cells were evaluated by flow cytometry and trypan blue staining. After 14 days, purity of NK cells was about 98% with more than 100 fold expansions compared with isolated NK cells (Figure 1). 

The cytotoxic effect of NK-92MI cell line (Shi et al., 2016) and also expanded NK cells using irradiated HFWT cells (Peng et al., 2004) or genetically modified K562 feeder cells (Kamiya et al., 2016) against target HCC cells were reported in previous studies. Even though these methods are very efficient, but as they were cancer cell-based approaches, therefore, they can be dangerous if any viable tumoric cells have been mixed with the expanded NK cells. In this study, we investigated whether expansion of NK cells using autologous irradiated PBMC as feeder layer with` OKT3 could boost the antitumor properties of fresh isolated NK cells against human HCC cells. So, the efficacy and cytotoxicity of iNKC and eNKC agains target HCC cells were evaluated through several methods. The proliferative index of HepG2 cells which exposed to NK cells was measured by CCK-8 kit. As illustrated in Figure 2, not only the viability of HCC cells was significantly decreased after coculture with different E:T cell count ratios, also the antitumor effect of eNKC was remarkably more than iNKC effect. Two main effector functions of NK cells against tumor cells are direct cytotoxic effect by releasing cytoplasmic granules (Perforin and Granzyme) and cytokine secretion such as IFN-γ and TNF-α. As reported in the previous studies, the *CD107a* expression is known as a marker of degranulation of cytotoxic NK cells after stimulation with target cells and correlates closely with antitumor effect of NK cells (Alter et al., 2004). The cytokines secreted from activated NK cells, in particular IFN-γ, perform critical functions in cancer surveillance, antiviral defense and antitumor responses of NK cells (Street et al., 2001; Schroder et al., 2004). Thus, we examined the functional activity of expanded NK cells under specific condition by evaluation of* CD107a *expression, secretion of TNF-α and IFN-γ and the expression level of Perforin and Granzyme against target cancer cells. The results of flow cytometry analysis showed significant induction of* CD107a* marker expression on the eNKC compared with iNKC or Ctrl group, after simulation with target HCC cells (Figure 3). In addition, measuring the content of TNF-α and IFN-γ in the supernatant of cocultured NK cells with HepG2 cells by ELISA assay, resulted in a significant increase in the eNKC treated group compared to iNKC or Ctrl group (Figure 4). As shown in Figure 5, the results of Real Time PCR analysis indicated that the expression levels of Perforin and Granzyme B in the eNKC after simulation with HepG2 cells were significantly much more than that of iNKC.

In conclusion, activation and expansion of NK cells using irradiated autologous PBMC as feeder layer with anti CD3 mAB, rhIL-2 and rh1L-15 without the use of cancer cells or other genetically modified feeder cells can be a very useful and effective method for suppression of HCC cancer cells. Overall, cell-to-cell communication and activator cytokines have crucial role for activation and proliferation of NK cells.

## Abbreviations

NK cell: Natural Killer cell

eNKC: expanded Natural Killer Cells

iNKC: isolated Natural Killer Cells

PBMC: Peripheral Blood Mononuclear Cells

HCC: Hepatocellular Carcinoma

IL: Interleukine

CCK-8: Cell Counting Kit-8

IFN: Interferon

TNF: Tumor Necrosis Factor

TRAIL: TNF-Related Apoptosis-Inducing Ligand

ADCC: Antibody-Dependent Cellular Cytotoxicity

MHC: Major Histocompatibility Complex

GVHD: Graft Versus Host Disease
